# Supplementary material for: Qualitative Analysis of a Home-Delivered Produce Prescription Intervention to Improve Food and Nutrition Security
Source: Nutrients. 2024 Nov 23;16(23):4010. doi: 10.3390/nu16234010 (PMC11643553; doi:10.3390/nu16234010)
Supplement: Supplementary file 1 [file nutrients-16-04010-s001.zip › Supplement S1_Interview Guide.pdf]

Supplement S1: Interview Guide Questions

1) Tell me about your overall experience with the FLiPRx program during the past year you participated.

2) Thinking back to when you were first told about this program, what were some of the things that made you interested in participating? A. a.
   a) **Probe**: What made you want to continue after the first 6 months?

3) In what ways do you feel the program influenced your families' eating habits?
   a) **Probe**: If you made changes, what changes do you intend to continue now that the program is over?
   b) **Probe**: What kinds of things might get in the way of you being able to continue these habits?
   c) **Probe**: What things might help you continue these habits?

4) In what ways do you feel the program changed your families' food purchasing habits?
   a) **Probe**: How has the program impacted your habits over the year around the other foods you buy? OR how you purchased other foods?
   b) **Probe**: If you had saved money on food, how did you use any money you saved on food?

5) Think about the nutrition education component of the program which included 3 things: #1. the printed recipe and skill cards you received in your produce box every other week, #2. the YouTube FLiP Tip videos sent by text every other week, and #3. the monthly online virtual "cooking class" called Ask The Chef. In general, what were your thoughts about these educational components?
   a) **Probe**: Which of these things did you like the most? and why?
   b) **Probe**: Which did you like the least? and why?

6) Approximately how many of the 26 FLiP Tip videos did you watch?
   a) **Probe**: What factors made you choose to watch or not watch a video and what would have made you more likely to watch more videos?
   b) **Probe**: In general, how do you like to receive valuable nutrition information for your family?

7) Approximately how many monthly classes (Ask The Chef) did you attend? (i.e. Only offered 1st Wednesday evening of each month)
   a) **Probe**: What would have made you more likely to attend more cooking classes?

8) Let's discuss the produce boxes in more detail. How did you use the food provided in the box? Probe: Did you try any new foods that were included in the produce box? If not, why?
   a) **Probe**: How did you use the produce box for your youngest children less than 5yo?
   b) **Probe**: Did you use most of the produce in the box? If any, what did you have to throw away?
   c) **Probe**: Did you share with family or neighbors, did you use it for celebrations, did you use it for snacks?

9) Prior to participating in this program, what was it like for you to get healthy and affordable food for your family? For example, some families might have trouble finding the types of foods (fruits and vegetables) you got during the program.
   a) **Probe**: In what ways has this program helped you overcome any of those challenges?

10) Knowing the program is coming to an end, have you thought about ways you are going to continue to get healthy and affordable food for your family?

11) If you have used any nutrition assistance programs (like SNAP, WIC, or food pantries, etc.), how did your experience of the FLiPRx program compare to your experience with other nutrition assistance programs?
    a) **Probe**: What are your thoughts on the reliability of the FLiPRx program vs others?
    b) **Probe**: What are your thoughts on your ability to pick your foods, quality of the food or safety of the food we provided vs others?

12) During the past year there have been many changes from the federal government to support families in accessing more nutrition and healthy foods during the pandemic. Did any of these changes support your family and if so, please describe? (i.e. PEBT-school meal money on EBT card, SNAP amount increased, WIC restrictions lifted)
    a) **Probe**: Has anything changed since last time the FLiPRx team talked about your use of federal and local supplemental food/nutrition programs?

13) Would you recommend this program to another family and why?
    a) **Probe**: Why should they participate?

14) We would like to consider improving this program for other families. Do you have any suggestions on how we can improve the program for our future families?

15) Is there anything else you would like to share with us about your experiences with the program?

16) Conclusion: Are you interested in further resources around accessing healthy affordable food?
